# Src/CK2/PTEN-Mediated GluN2B and CREB Dephosphorylations Regulate the Responsiveness to AMPA Receptor Antagonists in Chronic Epilepsy Rats

**DOI:** 10.3390/ijms21249633

**Published:** 2020-12-17

**Authors:** Ji-Eun Kim, Duk-Shin Lee, Hana Park, Tae-Cheon Kang

**Affiliations:** 1 Department of Anatomy and Neurobiology, College of Medicine, Hallym University, Chuncheon 24252, Korea; jieunkim@hallym.ac.kr (J.-E.K.); dslee84@hallym.ac.kr (D.-S.L.); M19050@hallym.ac.kr (H.P.); 2 Institute of Epilepsy Research, College of Medicine, Hallym University, Chuncheon 24252, Korea

**Keywords:** GRIA1, GYKI 52466, hippocampus, NMDA receptor, NR2B, perampanel, pilocarpine, refractory seizure

## Abstract

Both α-amino-3-hydroxy-5-methylisoxazole-4-propionic acid receptor (AMPAR) and N-methyl-D-aspartate receptor (NMDAR) have been reported as targets for treatment of epilepsy. To investigate the roles and interactions of AMPAR and NMDAR in ictogenesis of epileptic hippocampus, we analyzed AMPAR antagonists (perampanel and GYKI 52466)-mediated phosphatase and tensin homolog deleted on chromosome 10 (PTEN) regulation and glutamate ionotropic receptor NMDA type subunit 2B (GluN2B) tyrosine (Y) 1472 phosphorylation in epilepsy rats. Both perampanel and GYKI 52466 increased PTEN expression and its activity (reduced phosphorylation), concomitant with decreased activities (phosphorylations) of Src family-casein kinase 2 (CK2) signaling pathway. Compatible with these, they also restored the upregulated GluN2B Y1472 and Ca^2+^/cAMP response element-binding protein (CREB) serine (S) 133 phosphorylations and surface expression of glutamate ionotropic receptor AMPA type subunit 1 (GRIA1) to basal level in the epileptic hippocampus. These effects of perampanel and GYKI 52466 are observed in responders (whose seizure activities are responsive to AMPAR antagonists), but not non-responders (whose seizure activities were uncontrolled by AMPAR antagonists). Therefore, our findings suggest that Src/CK2/PTEN-mediated GluN2B Y1472 and CREB S133 regulations may be one of the responsible signaling pathways for the generation of refractory seizures in non-responders to AMPAR antagonists.

## 1. Introduction

Epilepsy is a neurological disorder, which is characterized by the spontaneous seizure episodes due to abnormal neuronal hyperactivities, with the prevalence of 6–8 per 1000 persons. The annual cumulative incidence of epilepsy is 67.77 per 100,000 persons, and the incidence rate is 61.44 per 100,000 person-years [1]. Despite its complex etiology, temporal lobe epilepsy (TLE, the most common refractory epilepsy) is often associated with an adverse developmental outcome and a widespread irreversible damage of the entorhinal cortex and the hippocampus [2]. Given the partial or total inefficacy of the conventional anti-epileptic drug (AED) treatment and the unrelenting nature of this form of epilepsy [1,2], the need for novel effective treatments is undeniable.

Although the underlying mechanisms of pharmacoresistant epilepsy are still unknown, several potential factors concerning refractory seizures have been proposed: (1) Over-expression or hyper-activation of drug efflux transporter (including ATP-cassette-binding protein) [3], (2) sustained releases of pro-inflammatory factors such as interleukin-1β in epileptic foci [4], (3) dysfunctions of channel/transporters including voltage-gated K^+^, Na^+^, Ca^2+^, or Cl^−^ channels or various ion/neurotransmitter transporters [5,6], (4) aberrant neural networks [6], and (5) maladaptive regulations of synaptic γ-aminobutyric acid (GABA)_A_ and glutamate receptors [7]. Therefore, multifactorial events are involved in the generation of refractory seizures, and more studies are needed to elucidate the molecular pathways concerning the pharmacoresistances for improvement of epilepsy therapies.

Glutamate-mediated neuronal hyperexcitation plays a causative role in eliciting the pathogenesis of epilepsy and is a decisive factor in the secondary neuronal damage induced by seizures. Thus, the regulation of glutamate receptor activities is one of the major therapeutic targets to inhibit the seizure generation (ictogenesis). Among glutamate receptors, *N*-methyl-d-aspartate receptor (NMDAR), α-amino-3-hydroxy-5-methylisoxazole-4-propionic acid receptor (AMPAR) and kainate receptor (KAR) have been reported as targets for treatment of epilepsy [8]. Furthermore, the interactions of NMDAR and AMPAR play an important role in synaptic plasticity in the normal brain. Briefly, NMDAR activation affects glutamate ionotropic receptor AMPA type subunit 1 (GRIA1) phosphorylation (S831 and S845) and trafficking into the synapses during inductions of long-term potentiation (LTP) and long-term depression (LTD) [9]. Repetitive AMPAR activations also result in sufficient depolarization to relieve Mg^2+^-mediated NMDAR inhibition and enable NMDAR-mediated excitation, which subsequently trigger synaptic plasticity [10,11]. Therefore, it is plausible that reciprocal AMPAR-NMDAR regulation would be involved in ictogenesis and steadily progressive seizure-related brain pathologic plasticity in the pathogenesis of epilepsy, although it remains as yet unknown.

NMDAR is hetero-tetrameric assemblies of a core glutamate ionotropic receptor NMDA type subunit 1 (GluN1, also referred as GRIN1 or NR1) subunit with different modulatory GluN2A-D subunits (so-called GRIN2A-D or NR2A-D) and less common GluN3 (GRIN3A-B or NR3A-B) [12,13]. The kinetics of NMDAR is much slower than that of AMPAR. In synaptic transmission, NMDAR activation is slow and prolonged due to the requirement for membrane depolarization [14]. However, the roles of NMDAR in ictogenesis remain controversial: A decrease of GluN1 and GluN2B protein levels is shown in epileptic patient and animal models [15,16], suggesting both NMDAR loss in degenerating neurons and adaptive modifications in response to seizures or to neuropathology. However, upregulations of GluN2B mRNA and its protein level are also found in non-sclerotic hippocampi and epileptic foci from epileptic patients [17,18]. In addition to expression level, composition, and localization of NMDAR subunits, the tyrosine (Y) 1472 phosphorylation of GluN2B subunit by the Src tyrosine kinase family is a key factor for determining of the NMDAR function, by increasing channel permeability to Ca^2+^ and stabilizing the receptor at the post-synaptic density (PSD). Indeed, GluN2B Y1472 phosphorylation is involved in the seizure susceptibility and ictogenesis in various seizure animal models including TLE [19,20]. Furthermore, ifenprodil (a selective GluN2B antagonist) strongly reduces spontaneous seizure activity in a mouse TLE model [21]. Thus, GluN2B Y1472 phosphorylation is highly correlated with heightened NMDAR currents that contribute to epileptiform discharges, resulting in seizure aggravation, and it will be of interest to more specifically investigate the underlying mechanisms of GluN2B Y1472 phosphorylation.

On the other hand, AMPAR is a tetramer composed of four types of subunit (GRIA1-4, so-called GluA1-4 or GluR1-4), and can be homo- or hetero-tetrameric. The GRIA2 subunit is the determinant of Ca^2+^ permeability: GRIA2-containing AMPAR is Ca^2+^ impermeable, but GRIA2-lacking AMPAR is Ca^2+^ permeable that is increased in chronic epileptic patients [16]. As compared to NMDAR, synaptic AMPAR turnover is continuous and fast [22,23,24]. Recent studies report that AMPAR antagonists terminate status epilepticus (SE, a prolonged seizure activity) in animal model, but NMDAR antagonists do not [25,26,27]. In addition, perampanel (a non-competitive AMPA receptor antagonist) results in an improvement of uncontrolled focal-onset seizure control as an add-on treatment. Thus, perampanel is an AED approved for the treatment of focal seizures and primary generalized tonic-clonic seizures in patients >12 years old [28]. In animal study, perampanel (2 mg/kg) effectively increases the after-discharge threshold in a rapid kindling model using immature rats, although it shows no effect on the number of stage 4–5 seizures in mature rats (postnatal 60 days) [29]. Furthermore, perampanel (6 mg/kg) effectively ceases LiCl-pilocarpine SE in rats [30]. Perampanel (8 mg/kg) also effectively inhibits spontaneous seizure activity in epileptic rats generated by LiCl-pilocarpine method [31]. In rat amygdala-kindling model, perampanel significantly increases the after-discharge threshold, and decreases the after-discharge duration and seizure severity at 50% higher intensity than the after-discharge threshold current at the dose of 10 mg/kg only, although the median toxic dose (TD_50_) of perampanel in rats is 9.14 mg/kg in rotarod test [32]. GYKI 52,466 (10 mg/kg) is another non-competitive AMPAR negative allosteric modulator, which shows effective anti-convulsive effects in a number of epilepsy models. However, GYKI 52,466 debilitates motor and cognitive side effects have been routinely documented at doses over 20 mg/kg [33,34]. Thus, it is likely that AMPAR antagonists may be more effective to inhibit spontaneous seizure activity than NMDAR antagonists. Furthermore, AMPAR antagonists (perampanel and GYKI 52,466) decrease GRIA1 surface expression in the epileptic rat hippocampus, which requires the restoring expression/activity of phosphatase and tensin homolog deleted on chromosome ten (PTEN) that drives depression of AMPAR-mediated synaptic responses following NMDAR activation [35,36]. Since PTEN performs GluN2B Y1472 dephosphorylation [37] and perampanel ameliorates NMDAR-mediated neuronal damage induced by ischemia [38], it is postulated that AMPAR antagonism affects NMDAR functions by regulating PTEN-mediated GluN2B Y1472 dephosphorylation, which is elusive.

Pilocarpine model (including LiCI-pilocarpine model) serves as a reliable animal model of the intractable epilepsy. The profiles of spontaneous recurrent seizures in this model resemble those of human TLE that is the most common form of drug-refractory epilepsy. Indeed, the lesions of mesial temporal structures including a well-known hippocampal sclerosis in human patients are similarly observed in this model [35,39]. To investigate the possible anti-epileptic effects of AMPAR antagonists, therefore, we analyzed AMPAR antagonists-mediated PTEN regulation and GluN2B Y1472 phosphorylation in responders (whose seizure activities are responsive to AMPA antagonists) and non-responders (whose seizure activities were uncontrolled by AMPAR antagonists) of LiCl-pilocarpine epilepsy rat model.

## 2. Results

### 2.1. Effects of AMPAR Antagonists on Chronic Spontaneous Seizure Activity

**Figure 1:**
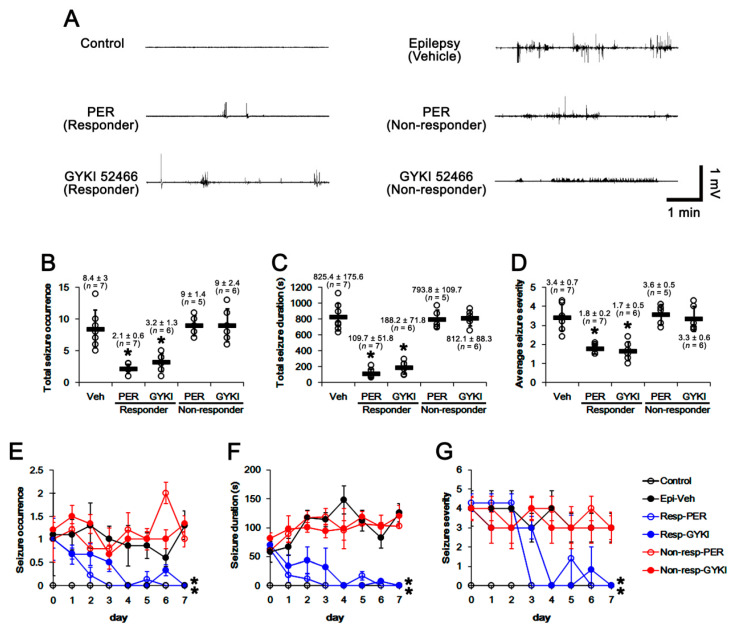
The effects of perampanel (PER) and GYKI 52,466 (GYKI) on spontaneous seizure activities in chronic epilepsy rats. Both α-amino-3-hydroxy-5-methylisoxazole-4-propionic acid receptor (AMPAR) antagonists effectively attenuate spontaneous seizure activities in responders. (**A**) representative electroencephalograms (EEG) in each group at two days after treatment. (**B**–**D**) Quantitative analyses of total seizure occurrence (**B**), total seizure duration (**C**) and average behavioral seizure score (seizure severity, D) in a seven-day period. Open circles indicate each individual value. Horizontal bars indicate mean value. Error bars indicate SD (* p < 0.05 vs. vehicle (Veh)-treated animals; Mann Whitney U-test for total seizure occurrence and average seizure severity; Student *t*-test for total seizure duration). (**E**–**G**) Quantitative analyses of the chronological effects of AMPAR antagonists on seizure occurrence (**E**), seizure duration (**F**), and seizure severity (**G**) over a seven-day period. Error bars indicate SD (* p < 0.05 vs. vehicle (Veh)-treated animals; Friedman test for seizure occurrence and seizure severity; repeated measures ANOVA for seizure duration).

In epileptic rats, the total seizure occurrence (number of seizures), the total seizure duration (the overall time spent in convulsive and non-convulsive seizures) on electroencephalograms (EEG), and average seizure severity (behavioral seizure core) were 8.4 ± 3, 825.4 ± 175.6 s, and 3.4 ± 0.7 in a seven-day period, respectively (n = 7, Figure 1A–D, Table S1). Five out of 12 rats in the perampanel-treated group were identified as non-responders to perampanel whose seizure activities were uncontrolled (total seizure occurrence, 9 ± 1.4; total seizure duration, 793.8 ± 109.7 s; average seizure severity, 3.6 ± 0.5, Figure 1A–D, Table S1). In responders (showing the significant reduction in seizure activities), the total seizure occurrence was 2.1 ± 0.6 (Z = 3.17, *p* = 0.001 vs. vehicle, Mann Whitney U-test, n = 7, respectively; Figure 1A–D, Table S1), the total seizure duration to 109.7 ± 51.8 s (*t*_(12)_ = 10.28, *p* < 0.001 vs. vehicle, Student *t*-test, n = 7, respectively; Figure 1A–D, Table S1), and average seizure severity was 1.8 ± 0.2 (Z = 3.15, *p* = 0.001 vs. vehicle, Mann-Whitney U-test, n = 7, respectively; Figure 1A–D, Table S1). Chronological analysis showed that perampanel gradually reduced seizure occurrence (χ^2^_(4)_ = 13.52, *p* = 0.009, Friedman test), seizure duration (F_(1,4)_ = 15.31, *p* = 0.017, repeated measures ANOVA), and seizure severity (χ^2^_(4)_ = 11.79, *p* = 0.019, Friedman test; Figure 1E–G) in responders over a seven-day period. Six out of 12 rats in the GYKI 52466-treated group were identified as non-responders (total seizure occurrence, 9 ± 2.4; total seizure duration, 812.1 ± 88.3 s; average seizure severity, 3.3 ± 0.6). In responders to GYKI 52466, the total seizure occurrence was 3.2 ± 1.3 (Z = 2.94, *p* = 0.001 vs. vehicle, Mann-Whitney U-test, n = 7 and 6, respectively; Figure 1A–D, Table S1), the total seizure duration was 188.2 ± 71.8 s (*t*_(11)_ = 8.18, *p* < 0.001 vs. vehicle, Student *t*-test, respectively; Figure 1A–D), and average seizure severity was 1.7 ± 0.5 (Z = 2.93, *p* = 0.001 vs. vehicle, Mann Whitney U-test, n = 7 and 6, respectively; Figure 1A–D). Chronological analysis also demonstrated that GYKI 52,466 also decreased seizure occurrence (χ^2^_(4)_ = 12.05, *p* = 0.017, Friedman test), seizure duration (F_(1,4)_ = 13.81, *p* = 0.021, repeated measures ANOVA), and the seizure severity (χ^2^_(4)_ = 13.06, *p* = 0.011, Friedman test; Figure 1E–G) in responders over a seven-day period.

### 2.2. Effects of AMPAR Antagonists on GluN2B Y1472 Phosphorylation

**Figure 2:**
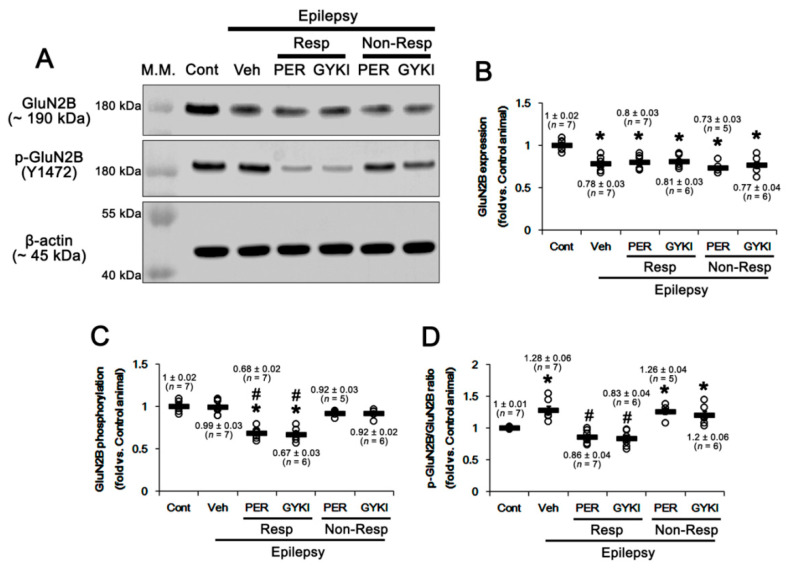
The effects of perampanel (PER) and GYKI 52,466 (GYKI) on total glutamate ionotropic receptor NMDA type subunit 2B (GluN2B) expression and its tyrosine (Y) 1472 phosphorylation (p-GluN2B) in chronic epilepsy rats. (**A**) Representative images for Western blot of GluN2B and GluN2B Y1472 levels in the hippocampal tissues. (**B**–**D**) Quantifications of GluN2B (**B**), p-GluN2B (**C**), and p-GluN2B/GluN2B ratio (**D**) in the hippocampal tissues. Open circles indicate each individual value. Horizontal bars indicate mean value. Error bars indicate SEM (*, # *p* < 0.05 vs. control and vehicle (Veh)-treated animals, respectively; one-way ANOVA with *post hoc* Bonferroni’s multiple comparison).

Since GluN2B Y1472 phosphorylation is involved in the seizure susceptibility and ictogenesis [19,20,21], we investigated the effects of AMPAR antagonists on its level in the epileptic hippocampus. As compared to control animals, GluN2B protein level was 0.78 ± 0.03-fold of control level in the epileptic hippocampus (*t*_(12)_ = 5.51, *p* < 0.001 vs. control animals, Student *t*-test; Figure 2A,B, Figure S1). Y1472 phosphorylation in the hippocampus was 0.99 ± 0.03-fold of control level (*t*_(12)_ = 0.14, *p* = 0.89 vs. control animals, Student *t*-test; Figure 2A,C, Figure S1). Thus, its phosphorylation ratio (phosphoprotein/total protein) was 1.28 ± 0.06-fold of control level (*t*_(12)_ = 4.7, *p* < 0.001 vs. control animals, Student *t*-test; Figure 2A,D, Figure S1). In responders to perampanel and GYKI 52466, GluN2B Y1472 phosphorylation levels were significantly reduced to 0.68 ± 0.02 and 0.67 ± 0.03-fold of control levels without affecting GluN2B protein levels, respectively (F_(2,17)_ = 37.5, *p* < 0.001 vs. vehicle, one-way ANOVA; Figure 2A,C, Figure S1). Thus, Y1472 phosphorylation ratios were 0.86 ± 0.04 and 0.83 ± 0.05-fold of control levels, respectively (F_(2,17)_ = 25.1, *p* < 0.001 vs. vehicle; Figure 2A,D, Figure S1). In non-responders to perampanel and GYKI 52466, GluN2B protein levels, Y1472 phosphorylation levels and its phosphorylation ratios were unaffected by each compound (Figure 2A–D, Figure S1). These findings indicate that AMPAR antagonists may inhibit GluN2B Y1472 phosphorylation in the epileptic hippocampus, which may ameliorate ictogenesis.

### 2.3. Effects of AMPAR Antagonists on PTEN Activity

GluN2B Y1472 site is dephosphorylated by PTEN [37]. Recently, we have reported that PTEN expression level in the epileptic hippocampus is lower than that in the control, and the anti-epileptic effects of AMPAR antagonists require the upregulations of its expression in responders [35]. However, we have not explored the effects of AMPAR antagonists on PTEN phosphoryaltion level in both responders and non-responders. Thus, we investigated whether PTEN expression/phosphorylation are relevant to GluN2B Y1472 phosphorylation in the epileptic hippocampi of responders and non-responders to AMPAR antagonists.

**Figure 3:**
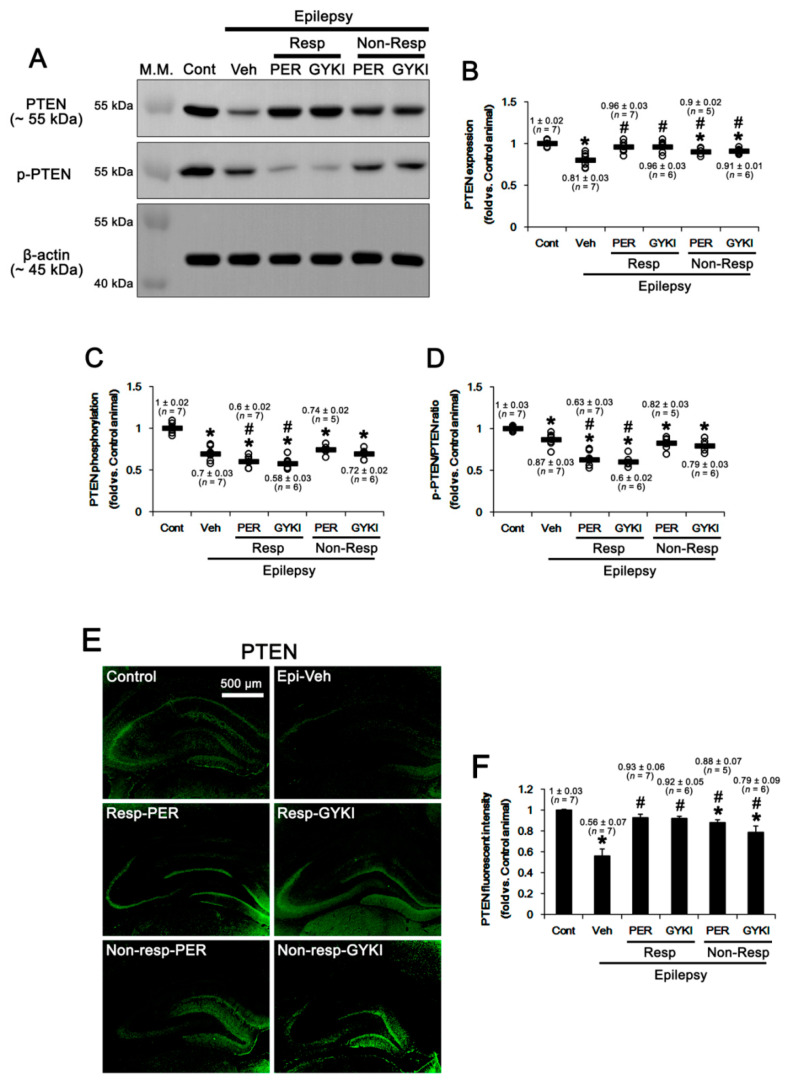
The effects of perampanel (PER) and GYKI 52,466 (GYKI) on total phosphatase and tensin homolog deleted on chromosome 10 (PTEN) expression and its phosphorylation (p-PTEN) in chronic epilepsy rats. Both AMPAR antagonists increase PTEN expression in both responders (Resp) and non-responders (Non-Resp) but decrease its phosphorylation only in responders. (**A**) Representative images for Western blot of PTEN and p-PTEN levels in the hippocampal tissues. (**B**–**D**) Quantifications of PTEN (**B**), p-PTEN (**C**) and p-PTEN/PTEN ratio (**D**) levels in the hippocampal tissues. Open circles indicate each individual value. Horizontal bars indicate mean value. Error bars indicate SEM (*, # *p* < 0.05 vs. control and vehicle (Veh)-treated animals, respectively; one-way ANOVA with post hoc Bonferroni’s multiple comparison). (**E**) Representative images for immunofluorescence of PTEN in the hippocampal tissues. (**F**) Quantifications of PTEN fluorescent intensity in the hippocampal tissues. Error bars indicate SEM (*, # *p* < 0.05 vs. control and vehicle (Veh)-treated animals, respectively; one-way ANOVA with post hoc Bonferroni’s multiple comparison).

In epileptic hippocampus, PTEN expression was 0.8 ± 0.03-fold of control level (*t*_(12)_ = 5.4, *p* < 0.001 vs. control animals, Student *t*-test; Figure 3A,B, Figure S2). In responders, perampanel and GYKI 52,466 increased PTEN expression to 0.96 ± 0.03-fold of control level in both groups (F_(2,17)_ = 9.3, *p* = 0.002 vs. vehicle, one-way ANOVA; Figure 3A,B, Figure S2). In non-responders, perampanel and GYKI 52,466 increased PTEN expression to 0.9 ± 0.02 and 0.91 ± 0.01-fold of control level, respectively (F_(2,15)_ = 5.6, *p* = 0.02 vs. vehicle, one-way ANOVA; Figure 3A,B, Figure S2). Their efficacies in non-responders were similar to those in responders (F_(3,20)_ = 1.7, *p* = 0.21, one-way ANOVA; Figure 3A,B, Figure S2), although PTEN expression levels were lower than those in control animals (F_(2,15)_ = 11.4, *p* = 0.001, one-way ANOVA; Figure 3A,B, Figure S2). PTEN phosphorylation level in epileptic hippocampus was 0.7 ± 0.03-fold of control level (*t*_(12)_ = 7.6, *p* < 0.001 vs. control animals, Student *t*-test; Figure 3A,C, Figure S2). In addition, PTEN phosphorylation ratio was 0.87 ± 0.03-fold of control level (*t*_(12)_ = 4.3, *p* = 0.001 vs. control animals, Student *t*-test; Figure 3A,D, Figure S2). In responders, perampanel and GYKI 52,466 reduced PTEN phosphorylation level to 0.6 ± 0.02 and 0.58 ± 0.03-fold of control level, respectively (F_(2,17)_ = 4.6, *p* = 0.03 vs. vehicle, one-way ANOVA; Figure 3A,C, Figure S2) and its ratio to 0.63 ± 0.03 and 0.6 ± 0.02-fold of control level, respectively (F_(2,17)_ = 22.3, *p* < 0.001 vs. vehicle, one-way ANOVA; Figure 3A,D, Figure S2). In non-responders, perampanel and GYKI 52,466 did not affect PTEN phosphorylation level (F_(2,15)_ = 0.6, *p* = 0.55 vs. vehicle, one-way ANOVA; Figure 3A,C, Figure S2) and its ratio (F_(2,15)_ = 1.5, *p* = 0.26; Figure 3A,D, Figure S2). Regarding phosphorylation-mediated inhibition of PTEN activity [40], it is likely that the reduced PTEN phosphorylation (the increased its activity) may be an adaptive response to the decreased expression in the epileptic hippocampus, while this alteration may be insufficient to suppress spontaneous seizure activities.

Immunofluorescent studies revealed that PTEN intensity was reduced to 0.56 ± 0.02-fold of control level in the epileptic hippocampus (*t*_(12)_ = 3.2, *p* = 0.008 vs. control animals, Student *t*-test; Figure 3E,F), due to massive neuronal death [35,39]. Perampanel and GYKI 52,466 up-regulated PTEN intensity to 0.93 ± 0.06 and 0.92 ± 0.05-fold of control level in responders, respectively (F_(2,17)_ = 6.18, *p* = 0.01 vs. vehicle, one-way ANOVA; Figure 3E,F). Both AMPAR antagonists also increased PTEN intensity to 0.88 ± 0.03 and 0.79 ± 0.06-fold of control level in non-responders, respectively (F_(2,15)_ = 3.91, *p* = 0.04 vs. vehicle, one-way ANOVA; Figure 3E,F). There was no difference in PTEN intensity between responders and non-responders (F_(3,20)_ = 2.18, *p* = 0.12, one-way ANOVA; Figure 3E,F). The increased PTEN intensities were mainly observed in dentate granule cells as well as remaining CA2-3 neurons (Figure 3E). Considering downregulation of GluN2B Y1472 phosphorylation in responders in the present study, our findings indicate that impaired PTEN-mediated GluN2B Y1472 dephosphorylation may play an important role in the generation of refractory seizures in response to AMPAR antagonists in chronic epilepsy rats.

### 2.4. AMPAR Antagonists-Mediated Inhibition of Src-CK2 Signaling Pathway in the Epileptic Hippocampus

**Figure 4:**
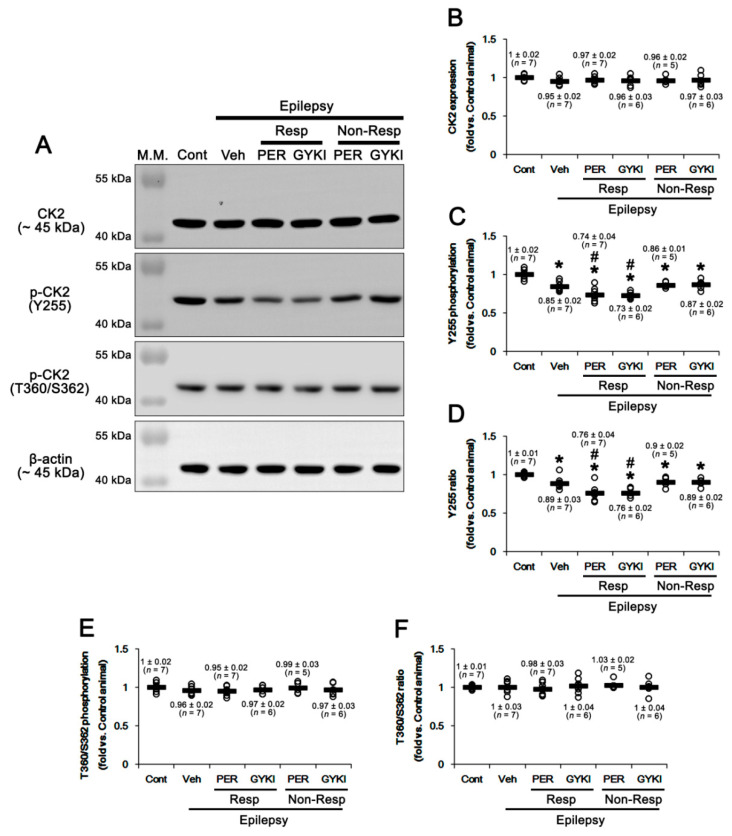
The effects of perampanel (PER) and GYKI 52,466 (GYKI) on total casein kinase 2 (CK2) and its Y255 and T360/S362 phosphorylations in chronic epilepsy rats. Both AMPAR antagonists reduce only CK2 Y255 phosphorylation in responders (Resp), but not non-responders (Non-Resp). (**A**) Representative images for Western blot of CK2 and p-CK2 levels in the hippocampal tissues. (**B**–**F**) Quantifications of CK2 (**B**), p-CK2 Y255 (**C**), p-CK2 Y255/CK2 ratio (**D**), p-CK2 T360/S362 (**E**), and p-CK2 T360/S362/CK2 ratio (**F**) levels in the hippocampal tissues. Open circles indicate each individual value. Horizontal bars indicate mean value. Error bars indicate SEM (*, # *p* < 0.05 vs. control and vehicle (Veh)-treated animals, respectively; one-way ANOVA with post hoc Bonferroni’s multiple comparison).

Casein kinase 2 (CK2)-mediated phosphorylations at serine (S) 370, S380, threonine (T) 382, T383, and S 385 sites inhibit PTEN stability and activity [41,42]. Thus, we explored whether AMPAR antagonists affect CK2 expression in the epileptic hippocampus. In the present study, CK2 protein level in the epileptic hippocampus was similar to that in the control, which was unaffected by AMPAR antagonists (*F*_(5,32)_ = 0.6, *p* = 0.72, one-way ANOVA; Figure 4A,B, Figure S3). CK2 is activated by phosphorylation of Y255 and T360/S362 sites, which are phosphorylated by Src family protein tyrosine kinases and extracellular signal-regulated kinase 1/2 (ERK1/2), respectively [43,44]. In the present study, CK2 Y255 phosphorylation level was 0.85 ± 0.02-fold of control level in the epileptic hippocampus (*t*_(12)_ = 4.6, *p* < 0.001, Student *t*-test; Figure 4A,C, Figure S3). Thus, CK2 Y255 phosphorylation ratio was 0.89 ± 0.03-fold of control level (*t*_(12)_ = 3.3, *p* = 0.006 vs. control animals, Student *t*-test; Figure 4A,D, Figure S3). In responders, perampanel and GYKI 52,466 further decreased the CK2 Y255 phosphorylation level to 0.74 ± 0.04 and 0.73 ± 0.02-fold of control level, respectively (*F*_(2,17)_ = 5.9, *p* = 0.01 vs. vehicle, one-way ANOVA; Figure 4A,C, Figure S3). Both AMPAR antagonists reduced Y255 phosphorylation ratios to 0.76 ± 0.04 and 0.76 ± 0.02-fold of control level (*F*_(2,17)_ = 5.1, *p* = 0.02 vs. vehicle, one-way ANOVA; Figure 4A,D, Figure S3). In non-responders, perampanel and GYKI 52,466 did not affect Y255 phosphorylation level (*F*_(2,15)_ = 0.3, *p* = 0.76 vs. vehicle, one-way ANOVA; Figure 4A,C, Figure S3) and its ratio (*F*_(2,15)_ = 0.05, *p* = 0.95, one-way ANOVA; Figure 4A,D, Figure S3). CK2 T360/S362 phosphorylation level (*t*_(12)_ = 1.4, *p* = 0.19, Student *t*-test; Figure 4A,E, Figure S3) and its phosphorylation ratio (*t*_(12)_ = 0.14, *p* = 0.89, Student *t*-test; Figure 4A,F, Figure S3) in the epileptic hippocampus were similar to those in controls. Both perampanel and GYK 52,466 did not influence CK2 T360/S362 phosphorylation level (*F*_(4,26)_ = 0.3, *p* = 0.85, one-way ANOVA; Figure 4A,E, Figure S3) and its phosphorylation ratio (*F*_(4,26)_ = 0.3, *p* = 0.89, one-way ANOVA; Figure 4A,F, Figure S3) in responders and non-responders. These findings indicate that AMPAR antagonists may increase PTEN activity by inhibiting CK2 Y255 phosphorylation in responders.

**Figure 5:**
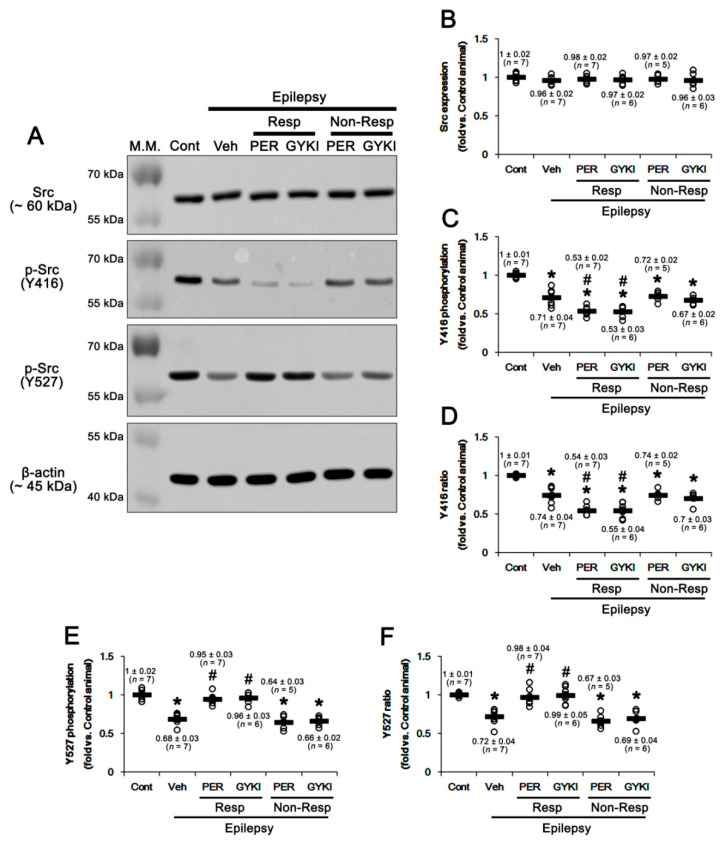
The effects of perampanel (PER) and GYKI 52,466 (GYKI) on total Src family (Src) and its Y416 and Y527 phosphorylations in chronic epilepsy rats. Both AMPAR antagonists reduce Src Y416 phosphorylation in responders (Resp), but not non-responders (Non-Resp), while they increase Src Y527 phosphorylation only in responders. (**A**) Representative images for Western blot of CK2 and p-CK2 levels in the hippocampal tissues. (**B**–**F**) Quantifications of Src (**B**), p-Src Y416 (**C**), p-Src Y416/Src ratio (**D**), p-Src Y527 (**E**), and p-Src Y527/Src ratio (**F**) levels in the hippocampal tissues. Open circles indicate each individual value. Horizontal bars indicate mean value. Error bars indicate SEM (*, # *p* < 0.05 vs. control and vehicle (Veh)-treated animals, respectively; one-way ANOVA with post hoc Bonferroni’s multiple comparison).

The Src family activities are reversely modulated by phosphorylation of two distinct tyrosine sites: Y416 autophosphorylation upregulates its kinase activity. In contrast, Y507 phosphorylation decreases its enzyme activity, although Y527 dephosphorylation is insufficient for full activation of Src [45,46]. Thus, we also investigated the effects of AMPAR antagonists on Src phosphorylations. Src protein level in the epileptic hippocampus was similar to that in the control, which was not influenced by AMPAR antagonists (*F*_(5,32)_ = 0.4, *p* = 0.85, one-way ANOVA; Figure 5A,B, Figure S4). Src Y416 phosphorylation level was 0.71 ± 0.04-fold in the epileptic hippocampus than that in controls (*t*_(12)_ = 6.7, *p* < 0.001 vs. control animals, Student *t*-test; Figure 5A,C, Figure S4) and its phosphorylation ratio was 0.74 ± 0.04-fold of control level (*t*_(12)_ = 6.2, *p* < 0.001 vs. control animals, Student *t*-test; Figure 5A,D, Figure S4). In responders, perampanel and GYKI 52,466 decreased Src Y416 phosphorylation level to 0.53 ± 0.02 and 0.53 ± 0.03-fold of control level, respectively (*F*_(2,17)_ = 10.3, *p* = 0.001 vs. vehicle, one-way ANOVA; Figure 5A,C, Figure S4). Both AMPAR antagonists reduced Y416 phosphorylation ratios to 0.54 ± 0.03 and 0.55 ± 0.04-fold of control level, respectively (*F*_(2,17)_ = 10.0, *p* = 0.001 vs. vehicle, one-way ANOVA; Figure 5A,D, Figure S4). In non-responders, perampanel and GYKI 52,466 did not affect Y416 phosphorylation level (*F*_(2,15)_ = 0.6, *p* = 0.56 vs. vehicle, one-way ANOVA; Figure 5A,C, Figure S4) and its ratio (*F*_(2,15)_ = 0.4, *p* = 0.67, one-way ANOVA; Figure 5A,D, Figure S4).

Similar to Y416 phosphorylation, Src Y527 phosphorylation level was decreased to 0.68 ± 0.03-fold of control level in chronic epilepsy rats (*t*_(12)_ = 8.6, *p* < 0.001 vs. control animals, Student *t*-test; Figure 5A,E, Figure S4). Y527 phosphorylation ratio was 0.72 ± 0.04-fold of control level (*t*_(12)_ = 6.9, *p* < 0.001, Student *t*-test; Figure 5A,F, Figure S4). In responders, perampanel and GYKI 52,466 increased Src Y527 phosphorylation level to 0.95 ± 0.03 and 0.96 ± 0.03-fold of control level, respectively (*F*_(2,17)_ = 30.1, *p* < 0.001 vs. vehicle, one-way ANOVA). Both AMPAR antagonists also elevated its phosphorylation ratio to 0.97 ± 0.04 and 0.99 ± 0.05-fold of control level (*F*_(2,17)_ = 12.8, *p* < 0.001 vs. vehicle, one-way ANOVA), respectively (Figure 5A,E,F, Figure S4). In non-responders, perampanel and GYKI 52,466 did not affect Y527 phosphorylation level (*F*_(2,15)_ = 0.5, *p* = 0.62 vs. vehicle, one-way ANOVA; Figure 5A,E, Figure S4) and its ratio (*F*_(2,15)_ = 0.5, *p* = 0.62, one-way ANOVA; Figure 5A,F, Figure S4). These findings indicate that AMPAR antagonists may inhibit Src activity in responders, which would decrease CK2 kinase activity.

### 2.5. AMPAR Antagonists-Mediated Inhibition of CREB Phosphorylation

**Figure 6:**
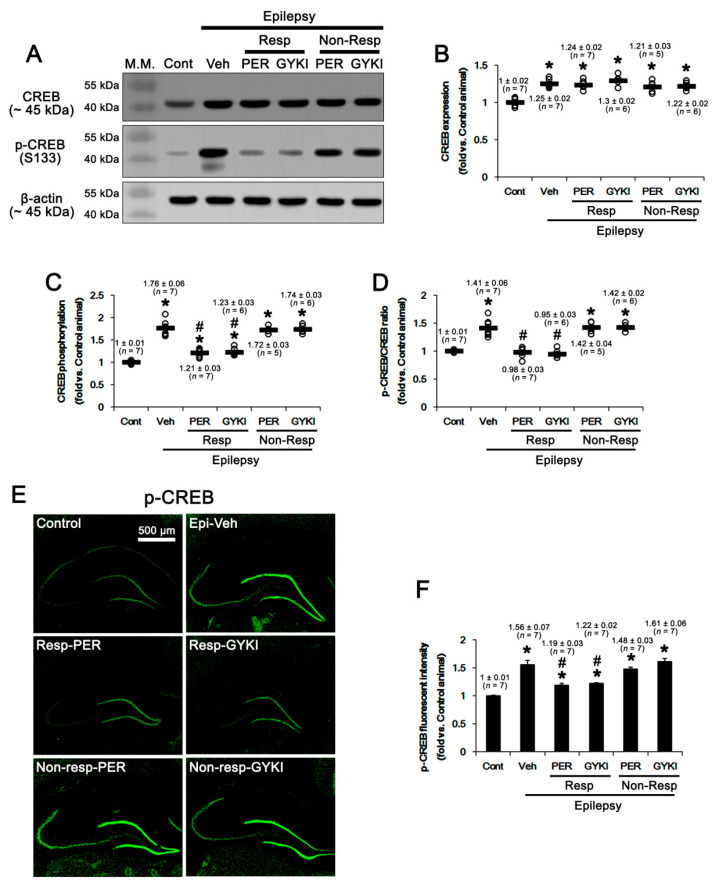
The effects of perampanel (PER) and GYKI 52,466 (GYKI) on total Ca^2+^/cAMP response element-binding protein (CREB) and its S133 phosphorylation in chronic epilepsy rats. Both AMPAR antagonists reduce CREB S133 phosphorylation in responders (Resp), but not non-responders (Non-Resp). (**A**) Representative images for Western blot of CREB and p-CREB levels in the hippocampal tissues. (**B**–**F**) Quantifications of CREB (**B**), p-CREB S133 (**C**), and p-CREB S133 ratio (**D**) levels in the hippocampal tissues. Open circles indicate each individual value. Horizontal bars indicate mean value. Error bars indicate SEM (*, # *p* < 0.05 vs. control and vehicle (Veh)-treated animals, respectively; one-way ANOVA with post hoc Bonferroni’s multiple comparison).

Ca^2+^/cAMP response element-binding protein (CREB) is necessary for the maintenance of GRIA1 in the postsynaptic density (PSD) of hippocampal neurons under basal conditions [47]. Interestingly, CREB activity increases in experimental and human TLE. Furthermore, decreased CREB activity can suppress spontaneous seizures and reduce the duration of SE in a rodent model of epilepsy [48,49]. The transcription activity of CREB is regulated by phosphorylation at S133 site [50,51]. Recently, it has been reported that AMPAR antagonism decreases CREB phosphorylation ratio in the hippocampus of morphine-abused animals [52]. Since PTEN negatively regulates CREB activity by dephosphorylating S133 site [37], it is likely that AMPAR antagonists-induced PTEN upregulation would reduce CREB activity in the epileptic hippocampus. In the present study, CREB protein level in the epileptic hippocampus was 1.25 ± 0.02-fold of control level (*t*_(12)_ = 8.7, *p* < 0.001 vs. control animals, Student *t*-test; Figure 6A,B, Figure S5), which was uninfluenced by AMPAR antagonists (*F*_(4,26)_ = 1.7, *p* = 0.19, one-way ANOVA; Figure 6A,B, Figure S5). CREB S133 phosphorylation level was 1.76 ± 0.06-fold of control level (*t*_(12)_ = 11.6, *p* < 0.001 vs. control animals, Student *t*-test; Figure 6A,C, Figure S5) and its phosphorylation ratio was 1.41 ± 0.06-fold of control level in the epileptic hippocampus (*t*_(12)_ = 6.9, *p* < 0.001 vs. control animals, Student *t*-test; Figure 6A,D, Figure S5). In responders, perampanel and GYKI 52,466 decreased CREB S133 phosphorylation level to 1.21 ± 0.06 and 1.23 ± 0.03-fold of control level, respectively (*F*_(2,17)_ = 44.0, *p* < 0.001 vs. vehicle, one-way ANOVA; Figure 6A,C, Figure S5) without affecting upregulated CREB protein level (*F*_(2,17)_ = 1.7, *p* = 0.21 vs. vehicle, one-way ANOVA; Figure 6A,B, Figure S5). Both AMPAR antagonists reduced S133 phosphorylation ratios to 0.98 ± 0.03 and 0.95 ± 0.03-fold of control level (*F*_(2,17)_ = 34.7, *p* < 0.001 vs. vehicle, one-way ANOVA; Figure 6A,D, Figure S5). In non-responders, perampanel and GYKI 52,466 did not affect CREB protein level (*F*_(2,15)_ = 0.8, *p* = 0.48 vs. vehicle, one-way ANOVA; Figure 6A,B, Figure S5), S133 phosphorylation level (*F*_(2,15)_ = 0.2, *p* = 0.81 vs. vehicle, one-way ANOVA; Figure 6A,C, Figure S5), and S133 phosphorylation ratio (*F*_(2,15)_ = 0.02, *p* = 0.98, one-way ANOVA; Figure 6A,D, Figure S5). Immunofluorescent studies revealed that the increased p-CREB intensities were mainly observed in dentate granule cells as well as remaining CA2-3 neurons (Figure 6E). p-CREB intensity was increased to 1.56 ± 0.07-fold of control level in the epileptic hippocampus (*t*_(12)_ = 3.0, *p* = 0.011 vs. control animals, Student *t*-test; Figure 6E,F). Perampanel and GYKI 52,466 down-regulated p-CREB intensity to 1.19 ± 0.03 and 1.22 ± 0.02-fold of control level in responders, respectively (*F*_(2,17)_ = 6.18, *p* = 0.01 vs. vehicle, one-way ANOVA; Figure 6E,F). Both AMPAR antagonists did not change p-CREB intensity in non-responders (*F*_(2,15)_ = 1.96, *p* = 0.18 vs. vehicle, one-way ANOVA; Figure 6E,F). These findings indicate that PTEN-mediated CREB S133 dephosphorylation may be one of the signaling pathways for anti-epileptic effect of AMPAR antagonists.

### 2.6. AMPAR Antagonists-Mediated Inhibition of GRIA Surface Expression

**Figure 7:**
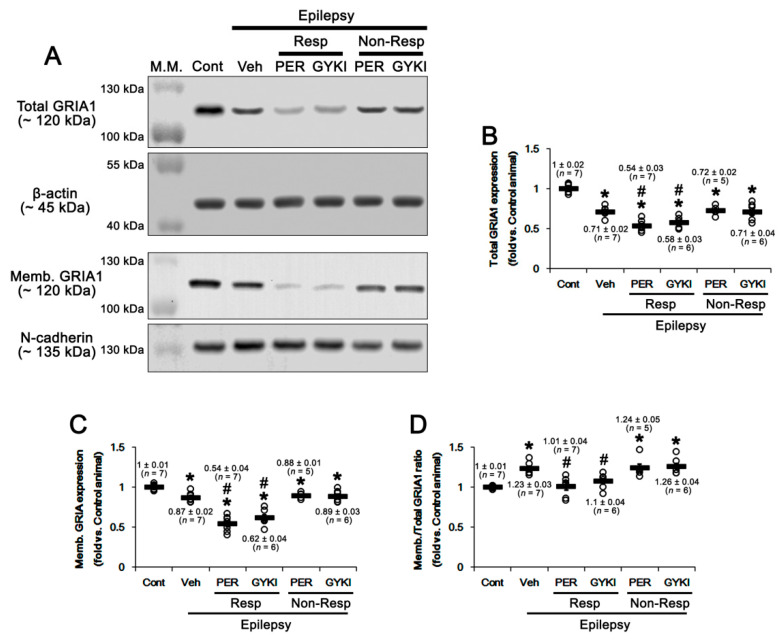
The effects of perampanel (PER) and GYKI 52,466 (GYKI) on total glutamate ionotropic receptor AMPA type subunit 1 (GRIA1) and its surface expression level in chronic epilepsy rats. Both AMPAR antagonists reduce total- and membrane GRIA1 expression in responders (Resp), but not non-responders (Non-Resp). (**A**) Representative images for Western blot of total- and membrane GRIA levels in the hippocampal tissues. (**B**–**D**) Quantifications of total GRIA1 (**B**), membrane GRIA1 (**C**), and membrane (memb.)/total GRIA1 ratio (**D**) levels in the hippocampal tissues. Open circles indicate each individual value. Horizontal bars indicate mean value. Error bars indicate SEM (*, # *p* < 0.05 vs. control and vehicle (Veh)-treated animals, respectively; one-way ANOVA with post hoc Bonferroni’s multiple comparison).

In a previous study [35], we have reported that AMPAR antagonists decrease GRIA1 surface expression in the epileptic rat hippocampus, which is abrogated by dipotassium bisperoxovanadium(pic) dihydrate (BpV(pic), a PTEN inhibitor). Considering the role of CREB in GRIA surface expression [47], it is plausible that PTEN-mediated CREB dephosphorylation may be relevant to the downregulation of GRIA1 induced by AMPAR antagonists. Consistent with our previous study [35], total GRIA1 protein level in the epileptic hippocampus was 0.71 ± 0.02-fold of control level (*t*_(12)_ = 9.3, *p* < 0.001 vs. control animals, Student *t*-test; Figure 7A,B, Figure S6), which was further reduced to 0.54 ± 0.03 and 0.58 ± 0.03-fold of control level in responders by perampanel and GYKI 52466, respectively (*F*_(2,17)_ = 12.7, *p* < 0.001 vs. vehicle, one-way ANOVA; Figure 7A,B, Figure S6). GRIA1 surface expression in the epileptic hippocampus was 0.87 ± 0.02-fold of control level (*t*_(12)_ = 4.9, *p* < 0.001 vs. control animals, Student *t*-test; Figure 7A,C, Figure S6), which was also decreased to 0.54 ± 0.04 and 0.62 ± 0.04-fold of control level in responders by perampanel and GYKI 52466, respectively (*F*_(2,17)_ = 25.7, *p* < 0.001 vs. vehicle, one-way ANOVA; Figure 7A,C, Figure S6). However, membrane/surface GRIA ratio in the epileptic hippocampus was increased to 1.23 ± 0.03-fold of control level (*t*_(12)_ = 7.8, *p* < 0.001 vs. control animals, Student *t*-test; Figure 7A,D, Figure S6). In responders, perampanel and GYKI 52,466 restored it to control level (*F*_(2,17)_ = 25.7, *p* < 0.001 vs. vehicle, one-way ANOVA; Figure 7A,D, Figure S6), but not in non-responders (*F*_(2,15)_ = 0.23, *p* = 0.8, one-way ANOVA; Figure 7A,D, Figure S6). These findings indicate that PTEN-mediated CREB S133 dephosphorylation induced by AMPAR antagonists may be involved in the downregulation of GRIA1 surface expression in the epileptic hippocampus.

## 3. Discussion

**Figure 8:**
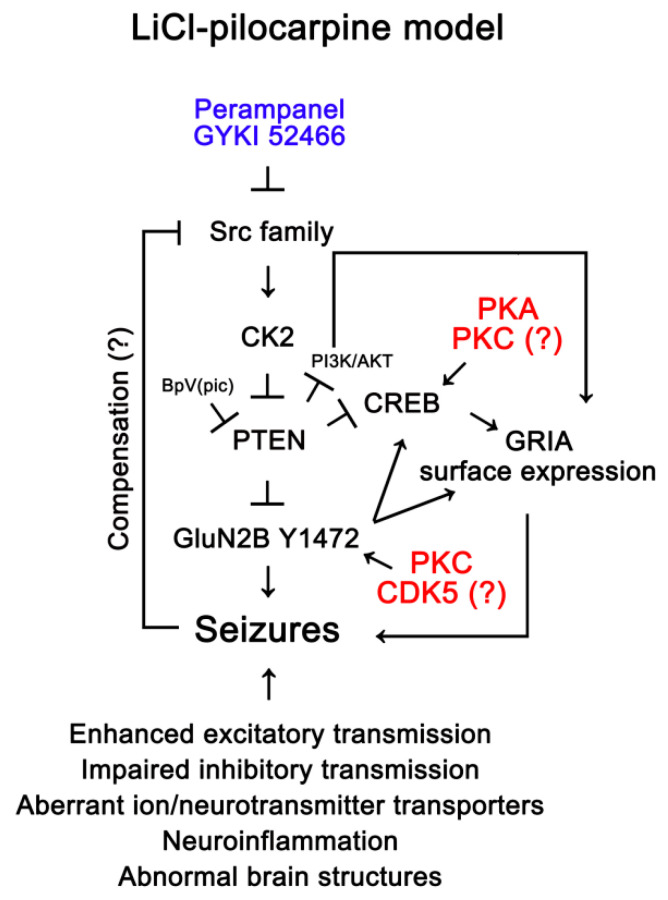
Scheme of the effects of AMPAR antagonists on spontaneous seizure activity in epilepsy rats based on a previous [35] and the present studies. In epilepsy rats, GluN2B Y1472 and CREB phosphorylations may be increased by multiple signaling pathways, such as protein kinase A (PKA), protein kinase C (PKC), and cyclin-dependent kinase 5 (CDK5). In addition, Src-CK2 axis may be inhibited as a compensatory response to the decreased PTEN expression via unknown mechanisms. However, this adaptive response may be insufficient to abrogate the hyper-phosphorylations of GluN2B and CREB. In responders, perampanel and GYKI 52,466 may further decrease Src-CK2 activity, which leads to increased PTEN activity (dephosphorylation) concomitant with its upregulation. Subsequently, the elevated PTEN activity may dephosphorylate GluN2B Y1472 and CREB S133 site that facilitate GRIA1 surface expression, concomitant with downregulation of phosphoinositide 3-kinase (PI3K)/AKT1 signaling pathway. These phenomena are not observed in non-responders and the dipotassium bisperoxovanadium(pic) dihydrate (BpV(pic), a PTEN inhibitor)-treated animals. Therefore, the present study demonstrates that this Src/CK2/PTEN-mediated GluN2B and CREB regulation may be required for the anti-epileptic effects of AMPAR antagonists.

The major findings in the present study are that the reduced PTEN expression was relevant to the upregulations of GluN2B Y1472 and CREB-S133 phosphorylations, which would be involved in ictogenesis in chronic epilepsy rats. However, a compensatory increase in PTEN activity was insufficient to suppress these phenomena and spontaneous seizure activity. Perampanel and GYKI 52,466 restored GluN2B Y1472 and CREB-S133 phosphorylation ratios to control level in responders, accompanied by the increased PTEN expression. In addition, both AMPAR antagonists decreased Src/CK2-mediated PTEN phosphorylation and GRIA1 surface expression. Thus, our findings suggest that Src/CK2/PTEN-mediated GluN2B Y1472 and CREB S133 regulations may be one of the responsible signaling pathways for the generation of refractory seizures in non-responders to AMPAR antagonists (Figure 8).

Y phosphorylations of GluN2B subunit enhance channel function of NMDAR [53]. In particular, Y1472 phosphorylation plays an important in the seizure susceptibility and ictogenesis of epilepsy [19,20,54]. In the present study, chronic epilepsy rats showed GluN2B Y1472 hyper-phosphorylation in the hippocampus, while its protein level was lower than that in controls. These results implicate that hyper-activation of NMDAR through Y1472 phosphorylation may be involved in the pathophysiological mechanisms of epileptic seizures. Unexpectedly, the present data demonstrate that perampanel and GYKI 52,466 effectively reduced Y1472 phosphorylation ratio in responders, but not non-responders. Since both perampanel and GYKI 52,466 have a minimal effect on NMDAR functions [32,55], these findings indicate that AMPAR antagonists may indirectly reduce GluN2B Y1472 phosphorylation via AMPAR-mediated signaling pathways rather than the direct binding to NMDAR. On the other hand, transient increase in GluN2B Y1472 phosphorylation by spontaneous seizures in epilepsy rats [56] raises the possibility that reduced Y1472 phosphorylation would be a secondary consequence from anti-convulsive effects of AMPAR antagonists. However, the present data demonstrate that perampanel and GYKI 52,466 diminished Src/CK2-mediated PTEN phosphorylation in responders, which negatively regulates GluN2B Y1472 phosphorylation [37]. Therefore, it is likely that at least in responders GluN2B Y1472 dephosphorylation may be an anti-ictogenic factor of AMPAR antagonists rather than a subsequent phenomenon.

Mutation or inactivation of PTEN contributes to seizure generation [57]. Indeed, we have recently reported that up-regulated nuclear factor-κB (NF-κB) activity diminished PTEN expression in the epileptic hippocampus [35]. Thus, it is likely that PTEN downregulation may be one of the ictogenic factors in the epileptic hippocampus. As well as expression level, phosphorylations also regulate PTEN stability and activity. In particular, CK2-mediated phosphorylation abrogates PTEN phosphatase activity [41,42], which increases GRIA1 surface expression [40]. Compatible with epilepsy patients [58], the present study reveals that Src family Y416 phosphorylation ratio was lower in the epileptic hippocampus than that in controls. CK2 Y255 (but not T360/S362 sites) phosphorylation level was also decreased in the epileptic hippocampus, as compared to controls. Since Src family and ERK1/2 phosphorylate CK2 Y255 and T360/S362 sites, respectively [43,44], our findings indicate that CK2-mediated PTEN phosphorylation in responders may be predominantly regulated by Src family rather than ERK1/2, although which kinase among the Src family is involved in this modulation remains to be resolved. Consistent with Src/CK2 phosphorylation levels, furthermore, PTEN phosphorylation ratio was lower in the epileptic hippocampus than that in controls. Considering the reduced PTEN expression in the epileptic hippocampus, it is likely that decreased Src/CK2-mediated PTEN phosphorylation may be an adaptive response to the reduced PTEN expression. In the present study, reduced PTEN expression in the epileptic hippocampus was restored by AMPAR antagonist in both responders and non-responders. In addition, AMPAR antagonists further diminished the ratios of PTEN, Src Y416, and CK2 Y255 phosphorylations and GRIA1 surface expression in responders, but not non-responders. Both AMPAR antagonists also restored the decreased Src Y527 phosphorylation level in responders, but not non-responders. Regarding the inhibitory effect of Y527 on Src family kinase activity [45,46], these findings indicate that AMPAR antagonists may inhibit Src family-mediated CK2 phosphorylation in responders, which would increase PTEN activity. Indeed, inhibitions of Src family and CK2 reduce GRIA1 phosphorylation and its surface expression [59,60], similar to the present data. Since PTEN inhibition by BpV(pic) abolishes anti-epileptic effects of AMPAR antagonists in responders [35], it is likely that dysregulations of PTEN-mediated GluN2B Y1472 dephosphorylation may be involved in the AMPAR antagonists-resistant seizures in chronic epilepsy rats. Taken together, our findings suggest that aberrant Src/CK2/PTEN phosphorylation may be one of the important signaling pathways to induce refractory seizures in response to AMPAR antagonists in chronic epilepsy rats.

CREB signaling pathway is involved in the pathogenesis and progression of epilepsy, since CREB expression is increased in animal models of epilepsy as well as in brain tissues of epilepsy patients [48,49]. Interestingly, the GluN2B gene contains Ca^2+^/cAMP response element (CRE) at the last 406–413 base pairs among the 5′-terminal mRNA sequence and GluN2B expression is prominently enhanced following CREB activation [61,62]. Thus, it is presumable that upregulated CREB phosphorylation (activity) would increase GluN2B expression level in the epileptic hippocampus. Consistent with previous studies [15,16], however, the present data show reduced GluN2B expression in epileptic rats. Instead, GluN2B Y1472 site was hyper-phosphorylated in the epileptic rats, as compared to controls, which was effectively decreased by AMPAR antagonists. These findings indicate that CREB activity may not be involved in the regulation of GluN2B expression level. Conversely, NMDAR activation rapidly increases CREB phosphorylation, which is abrogated by MK801 (an NMDAR antagonist) [63]. AMPAR also phosphorylates CREB S133 site mediated by activating Src tyrosine kinase [64]. Furthermore, an AMPAR inhibitor decreases CREB S133 phosphorylation ratio in the hippocampus [52]. Thus, it is likely that CREB hyper-phosphorylation may be a consequence from activations of NMDAR and/or AMPAR-mediated signaling pathways. Indeed, the present study demonstrates that AMPAR antagonists effectively reduced CREB phosphorylation (activity), but not its expression, in responders. Furthermore, valproic acid (VPA, the first-line drug for epilepsy treatment in clinical practice) also inhibits CREB activity by abrogating excessive CREB expression in children with epilepsy, as compared to that in healthy controls [65], although VPA does not affect CREB DNA binding activity in vitro [66]. Considering the role of CREB in the synaptic GRIA1 localization [47] and the inhibitory effects of AMPAR antagonists on GRIA surface expression [35], therefore our findings suggest that AMPAR antagonists-induced Src/CK2-medited PTEN activation may decrease CREB S133 phosphorylation, which would be involved in the reduced GRIA surface expression in responders.

On the other hand, the present data show that GluN2B Y1472 and CREB S133 phosphorylations were increased in the epileptic hippocampus, although Src, CK2, and PTEN phosphorylation were reduced (indicating increased PTEN activity). Regardless of PTEN activity, CREB phosphorylation is also regulated by the activation of multiple signaling cascades including protein kinase A (PKA), protein kinase C (PKC), and Ca^2+^-calmodulin-dependent protein kinase II (CAMKII) [67,68]. In addition, PKC and cyclin-dependent kinase 5 (CDK5) lead to GluN2B-Y1472 phosphorylation [69,70]. Indeed, activities of PKC and CDK5 are maintained or increased in the brain of epilepsy human patients and animal models [31,71]. Therefore, the enhanced CREB and GluN2B phosphorylations may result from the convergence of these signaling pathways, and the compensatory activation of PTEN, as aforementioned, may be insufficient to abrogate upregulation of these phosphorylations in the epileptic hippocampus of vehicle-treated animals and non-responders.

In the present study, we did not compare the pharmacokinetics of AMPAR antagonists between responders and non-responders. Thus, it could not be excluded the possibility that the decreased responsiveness of AMPAR antagonists in non-responders would be a consequence from the lower concentration of these compound induced by over-expression or hyper-activation of drug efflux transporters [3]. However, AMPAR antagonists restored the reduced PTEN expression in the hippocampus in both responders and non-responders at the same level, while they did not affect phosphorylations of GluN2B, Src, CK2, and PTEN in non-responders unlike responders. Since PTEN activity is required for the anti-epileptic effects of AMPAR antagonists [35], it is therefore likely that at least maladaptive regulation of Src/CK2/PTEN signaling pathway may be one of the important factors in pharmacoresistant seizures in response to AMPAR antagonists. Further studies are needed to elucidate the causes of the dysregulation of this pathway in non-responders.

## 4. Materials and Methods

### 4.1. Experimental Animals and Chemicals

Male Sprague—Dawley (SD) rats (7 weeks old) were housed under controlled conditions (22 ± 2 °C, humidity 55 ± 5%, a light-dark cycle on a 12-h on-off cycle) with ad libitum access to water and food throughout the experiments. All experimental protocols described below were approved by the Institutional Animal Care and Use Committee of Hallym University (#Hallym 2018-2, 26 April 2018 and #Hallym 2018-21, 8 June 2018). All reagents were obtained from Sigma-Aldrich (St. Louis, MO, USA), except as described.

### 4.2. Generation of Epileptic Rats

Rats were intraperitonieally (i.p.) given LiCl (127 mg/kg, i.p.) 24 h before pilocarpine injection (30 mg/kg, i.p.). To inhibit the peripheral effect of pilocarpine, atropine methylbromide (5 mg/kg i.p.) was pretreated 20 min before pilocarpine treatment. To control SE, diazepam (Valium; Hoffmann-la Roche, Neuilly-sur-Seine, France; 10 mg/kg, i.p.) was given 2 h after SE onset. Control animals received saline instead of pilocarpine. SE-experienced rats were video-monitored 8 h a day for 4 weeks for selecting chronic epilepsy rats that showed spontaneous seizure episodes [31,35,72]. Behavioral seizure scores were also evaluated according to Racine’s scale: 1. immobility, eye closure, twitching of vibrissae, sniffing, facial clonus; 2. head nodding associated with more severe facial clonus; 3. clonus of one forelimb; 4. rearing, often accompanied by bilateral forelimb clonus; and 5. rearing with loss of balance and falling accompanied by generalized clonic seizures. Animals showing behavioral seizures with Racine’s score ≥ 3 more than once were classified as epileptic rats.

### 4.3. Electrode Implantation

Epileptic rats (4 weeks after SE) were implanted with monopolar stainless-steel electrodes (Plastics One, Roanoke, VA, USA) in the right hippocampus under Isoflurane anesthesia (3% induction, 1.5–2% for surgery, and 1.5% maintenance in a 65:35 mixture of N_2_O:O_2_). Stereotaxic coordinates were −3.8 mm posterior; 2.0 mm lateral; −2.6 mm depth to bregma. Throughout surgery, the core temperature of each rat was maintained at 37–38 °C. The electrode was secured to the exposed skull with dental acrylic.

### 4.4. Drug Trials and Quantification of Seizure Activity

Three days after electrode implantation, baseline seizure activity was measured over 3 days. Thereafter, perampanel (2-(2-oxo-1-phenyl-5-pyridin-2-yl-1,2-dihydropyridin-3-yl)benzonitrile; 8 mg/kg, i.p., Eisai Korea Inc., Seoul, Korea), GYKI 52,466 (10 mg/kg, i.p.), or saline (vehicle) was administered daily at PM 6:00 over a 7-day period [31,35,72]. To select the responders and non-responders, and each dose was chosen as the maximum without adverse effects, based on previous studies [33,34,42,55]. EEG were detected with a DAM 80 differential amplifier (0.1–3000 Hz bandpass; World Precision Instruments, Sarasota, FL, USA) 2 h a day at the same time over a 7-day period. The data were digitized (1000 Hz) and analyzed using LabChart Pro v7 (ADInstruments, Bella Vista, New South Wales, Australia). Behavioral seizure severity was also evaluated according to Racine’s scale aforementioned. Non-responders were defined as showing no reduction in total seizure occurrence in a 7-day period, as compared with the pre-treatment stage. After recording (18 h after the last treatment), animals were used for Western blot.

### 4.5. Membrane Fraction and Western Blots

Eighteen hours after the last compound treatment, animals were sacrificed by decapitation, and the hippocampi were obtained. The hippocampal tissues were homogenized in lysis buffer (50 mM Tris containing 50 mM 4-(2-hydroxyethyl)-1-piperazineethanesulfonic acid (pH 7.4), ethylene glycol tetraacetic acid (pH 8.0), 0.2% Tergitol type NP-40, 10 mM ethylenediaminetetraacetic acid (pH 8.0), 15 mM sodium pyrophosphate, 100 mM β-glycerophosphate, 50 mM NaF, 150 mM NaCl, 2 mM sodium orthovanadate, 1 mM phenylmethylsulfonyl fluoride, and 1 mM dithiothreitol) containing protease inhibitor cocktail (Roche Applied Sciences, Branford, CT, USA) and phosphatase inhibitor cocktail (PhosSTOP^®^, Roche Applied Science, Branford, CT, USA). To investigate GluN2B expression and its Y1472 phosphorylation, 1% sodium dodecyl sulfate (SDS) was substituted for NP-40. Protein concentration determined using a Micro BCA Protein Assay Kit (Pierce Chemical, Rockford, IL, USA). To analyze GRIA1 surface expression, we used a subcellular Protein Fractionation Kit for Tissues (ThermoFisher Scientific Korea, Seoul, Korea), according to the manufacturer’s instructions. Western blot was performed by the standard protocol: Sample proteins (10 μg) were separated on a Bis-Tris sodium dodecyl sulfate-poly-acrylamide gel (SDS-PAGE) and transferred to membranes. Membranes were incubated with 2% bovine serum albumin (BSA) in Tris-buffered saline (TBS; in mM 10 Tris, 150 NaCl, pH 7.5, and 0.05% Tween 20), and then reacted with primary antibodies (Table 1) overnight at 4 °C. After washing, membranes were incubated in a solution containing horseradish peroxidase (HRP)-conjugated secondary antibodies for 1 h at room temperature. Immunoblots were detected and quantified using ImageQuant LAS4000 system (GE Healthcare Korea, Seoul, Korea). Optical densities of proteins were calculated with the corresponding amount of β-actin.

### 4.6. Immunohistochemstry

Animals were transcardially perfused with 4% paraformaldehyde under urethane anesthesia (1.5 g/kg i.p.), and after additional fixation for overnight at 4 °C. The brains were rinsed in PB containing 30% sucrose at 4 °C for 2 days. Thereafter, coronal sections (30 μm) were cut with a cryostat. After blockade with 10% goat serum (Vector, Burlingame, CA, USA) in PBS for 30 min at room temperature, slices were incubated with the primary antibody (Table 1) in PBS containing 0.3% triton X-100 at room temperature for overnight. After three washes in PBS, fluorescein isothiocyanate (FITC)-conjugated secondary antibodies (Vector, Burlingame, CA, USA) were applied for 1 h at room temperature. Brain sections incubated with preimmune serum or second antibody alone were used as negative controls. Ten hippocampal sections per each animal were randomly selected, and fluorescent intensity was measured using AxioVision Rel. 4.8 software (Carl Zeiss Korea, Seoul, Korea).

### 4.7. Data Analysis

Shapiro–Wilk *W*-test was used to evaluate the values on normality. Mann Whitney U-test (total seizure occurrence and average seizure severity) and Student’s *t*-test (comparison of total seizure duration and Western blot data) were applied to determine statistical significance of data. Comparisons among groups were also performed using repeated-measures ANOVA (chronological change in seizure duration), Friedman test (chronological changes in seizure occurrence and its severity) and one-way ANOVA followed by Bonferroni’s post hoc comparisons. A *p*-value less than 0.05 was considered to be significant.

## 5. Conclusions

In the present study, we obtained the following evidence using pilocarpine rat epilepsy model evolving to spontaneous recurrent seizures (Figure 8): Impairment of PTEN-mediated GluN2B Y1472 and CREB regulations may be one of the responsible signaling pathways for ictogenesis in chronic epilepsy rats. Perampanel and GYKI 52,466 may increase PTEN expression and elevate its activity by inhibiting Src-mediated CK2 phosphorylation. Increased PTEN activity by AMPAR antagonists diminished GluN2B Y1472 and CREB S133 phosphorylations that would enable increasing the surface GRIA1 expression. These effects of perampanel and GYKI 52,466 are observed in responders, but not non-responders. Thus, our findings suggest that and maladaptive regulation of Src/CK2/PTEN signaling pathway may contribute to the generation of refractory seizures in non-responders to AMPAR antagonists.

## Figures and Tables

**Table 1:** Primary antibodies used in the present study.

Antigen	Host	Manufacturer (Catalog Number)	Dilution Used
Casein kinase 2 (CK2)	Mouse	Millipore (#05-1431)	1:1000 (WB)
Ca^2+^/cAMP response element-binding protein (CREB)	Rabbit	Novus biologicals (NBP1-90364)	1:500 (WB)
glutamate ionotropic receptor NMDA type subunit 2B (GluN2B)	Rabbit	Thermo Scientific (OPA1-04022)	1:1000 (WB)
glutamate ionotropic receptor AMPA type subunit 1 (GRIA1)	Rabbit	Synaptic systems (#182011)	1:1000 (WB)
N-cadherin	Rabbit	Abcam (ab182030)	1:4000 (WB)
phospho (p)-CK2 T360/S362	Rabbit	Abcam (ab119410)	1:1000 (WB)
p-CK2 Y255	Rabbit	Invitrogen (#PA5-38831)	1:1000 (WB)
p-CREB S133	Rabbit	Novus biologicals (NB110-55727)	1:250 (IF) 1:5000 (WB)
p-GluN2B Y1472	Rabbit	BioVision (A2208-100)	1:1000 (WB)
p-phosphatase and tensin homolog deleted on chromosome 10 (PTEN)-S380/Y382/Y383	Rabbit	Cell signaling (#9549)	1:1000 (WB)
PTEN	Rabbit	Abcam (ab170941) Abcam (ab32199)	1:250 (IF) 1:10,000 (WB)
Src family	Rabbit	Cell signaling (#2108)	1:1000 (WB)
p-Src family Y416	Rabbit	Cell signaling (#6943)	1:1000 (WB)
p-Src family Y527	Rabbit	Cell signaling (#2105)	1:1000 (WB)
β-actin	Mouse	Sigma (#A5316)	1:5000 (WB)

IF, Immunofluorescence; WB, Western blot.

## Supplementary Materials

Supplementary Materials can be found at https://www.mdpi.com/1422-0067/21/24/9633/s1.

## Abbreviations

AMPAR	α-Amino-3-hydroxy-5-methyl-4-isoxazolepropionic acid receptor
CK2	casein kinase 2
CREB	Ca^2+^/cAMP response element-binding protein
NMDAR	*N*-methyl-d-aspartate receptor
PTEN	Phosphatase and tensin homolog deleted on chromosome ten
