# Supplementary material for: Src/CK2/PTEN-Mediated GluN2B and CREB Dephosphorylations Regulate the Responsiveness to AMPA Receptor Antagonists in Chronic Epilepsy Rats
Source: Int J Mol Sci. 2020 Dec 17;21(24):9633. doi: 10.3390/ijms21249633 (PMC7766850; doi:10.3390/ijms21249633)
Supplement: Supplementary file 1 [file ijms-21-09633-s001.pdf]

Supplementary information

Src/CK2/PTEN-mediated GluN2B and CREB dephosphorylations regulate the response to AMPA receptor antagonist in chronic epilepsy rats

Ji-Eun Kim[1,2], Duk-Shin Lee, Hana Park[1,2], and Tae-Cheon Kang[1,2*]

[1]Department of Anatomy and Neurobiology, College of Medicine, Hallym University, Chuncheon 24252, South Korea

[2]Institute of Epilepsy Research, College of Medicine, Hallym University, Chuncheon 24252, South Korea

* Correspondence to: T. -C. Kang, Department of Anatomy and Neurobiology, College of Medicine, Hallym University, Chuncheon, Kangwon-Do 24252, South Korea; Tel: +82-33-248-2524; Fax: +82-33-248-2525; E-mail: tckang@hallym.ac.kr

Supplementary Table 1. Average of weight and consumptions of food and water in each group

Group	Weight (g)	Food consumption (g/day)	Water consumption (ml/day)
Control	341 ± 28.1	32 ± 7.3	34 ± 5.5
Epilepsy (Vehicle)	317 ± 33.5	34 ± 8.1	28 ± 4.8
Responder (perampanel)	324 ± 19.3	29 ± 12.4	26 ± 7.9
Responder (GYKI 52466)	309 ± 21.8	27 ± 6.8	31 ± 3.8
Non-responder (perampanel)	298 ± 35.9	28. ± 9.4	28 ± 6.2
Non-responder (GYKI 52466)	301 ± 32.1	31. ± 7.7	32 ± 4.2

Mean ± SD; No statistical significance in each group.

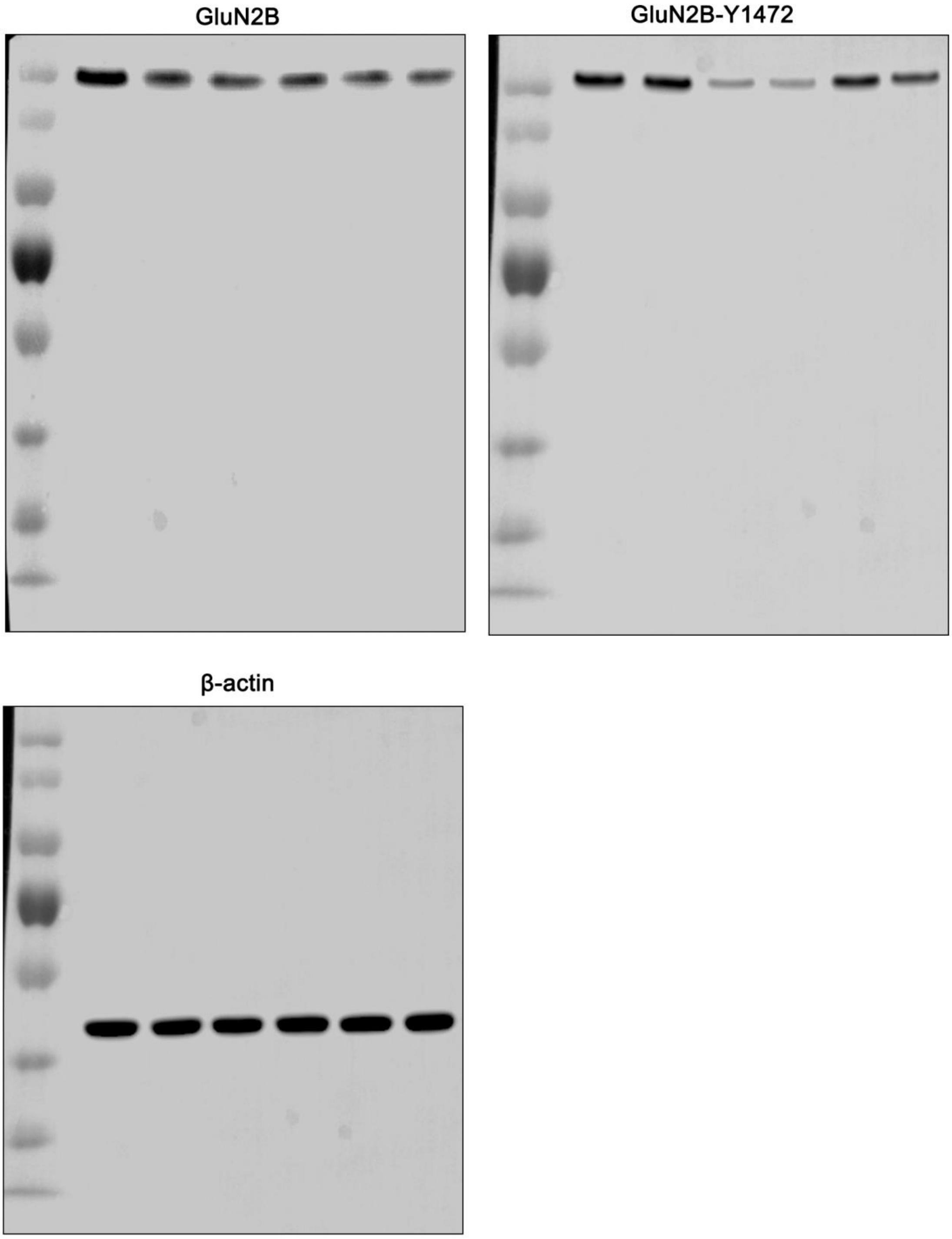

Supplementary Figure 1. Representative full-gel images of Western blots in Figure 2A.

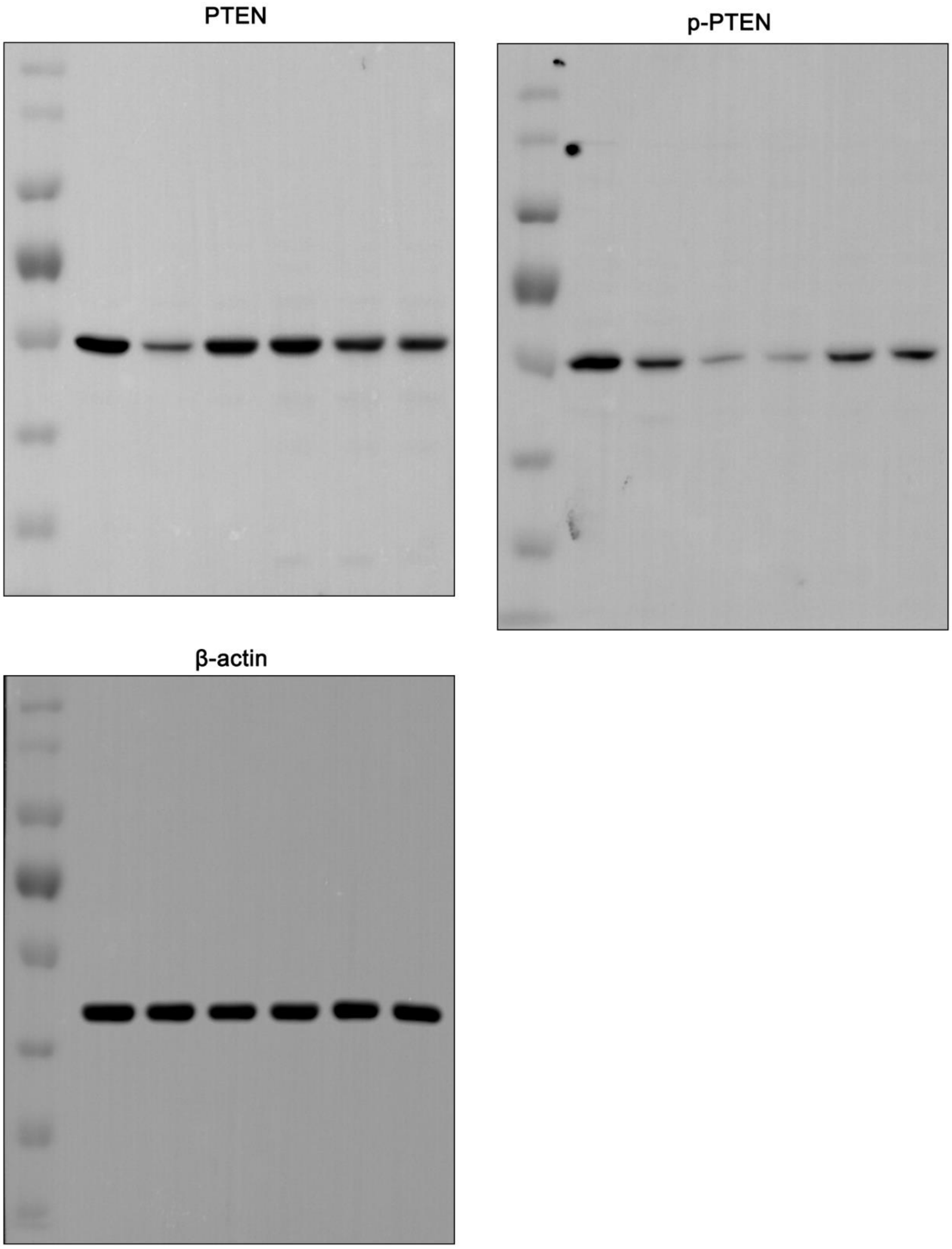

Supplementary Figure 2. Representative full-gel images of Western blots in Figure 3A.

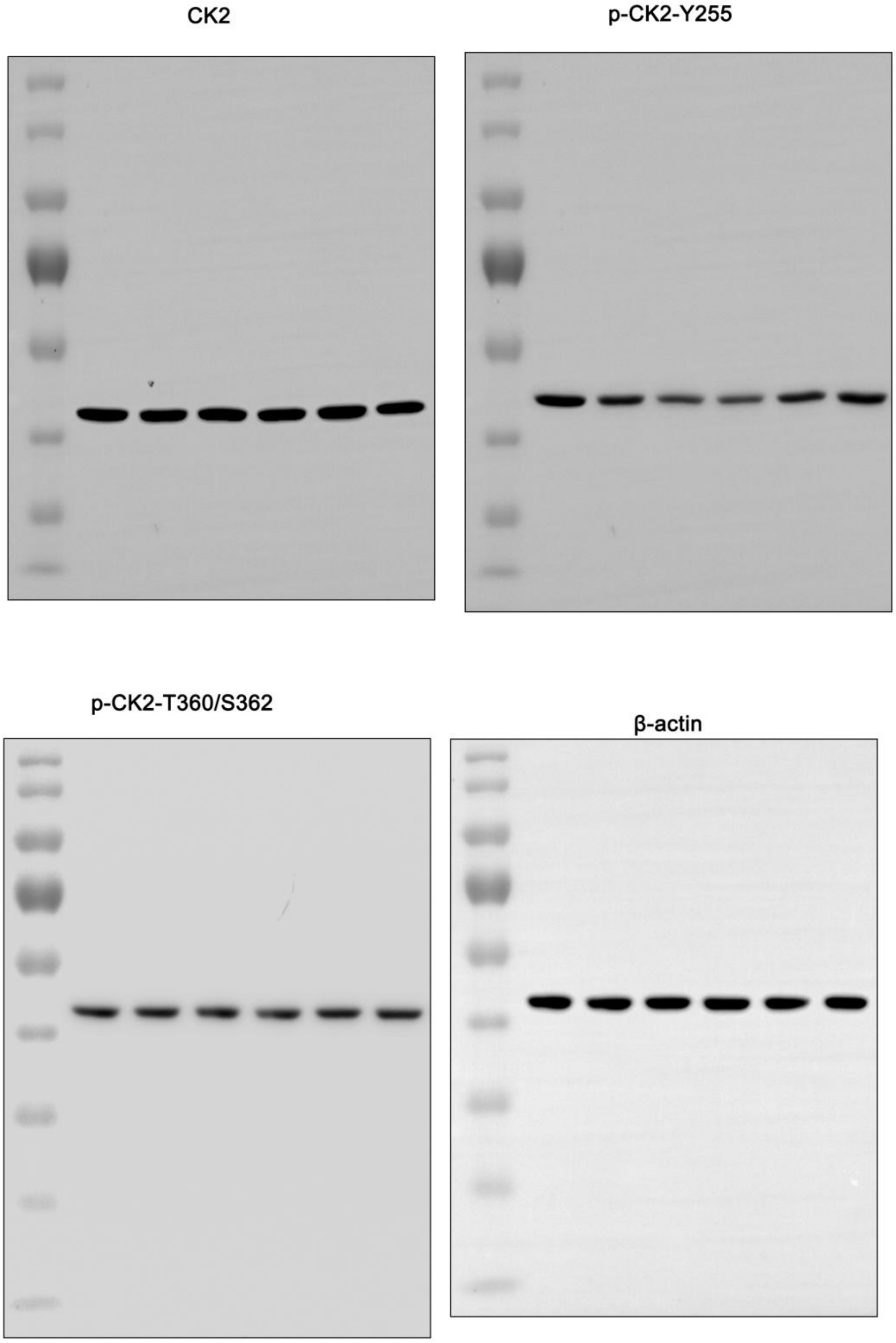

Supplementary Figure 3. Representative full-gel images of Western blots in Figure 4A.

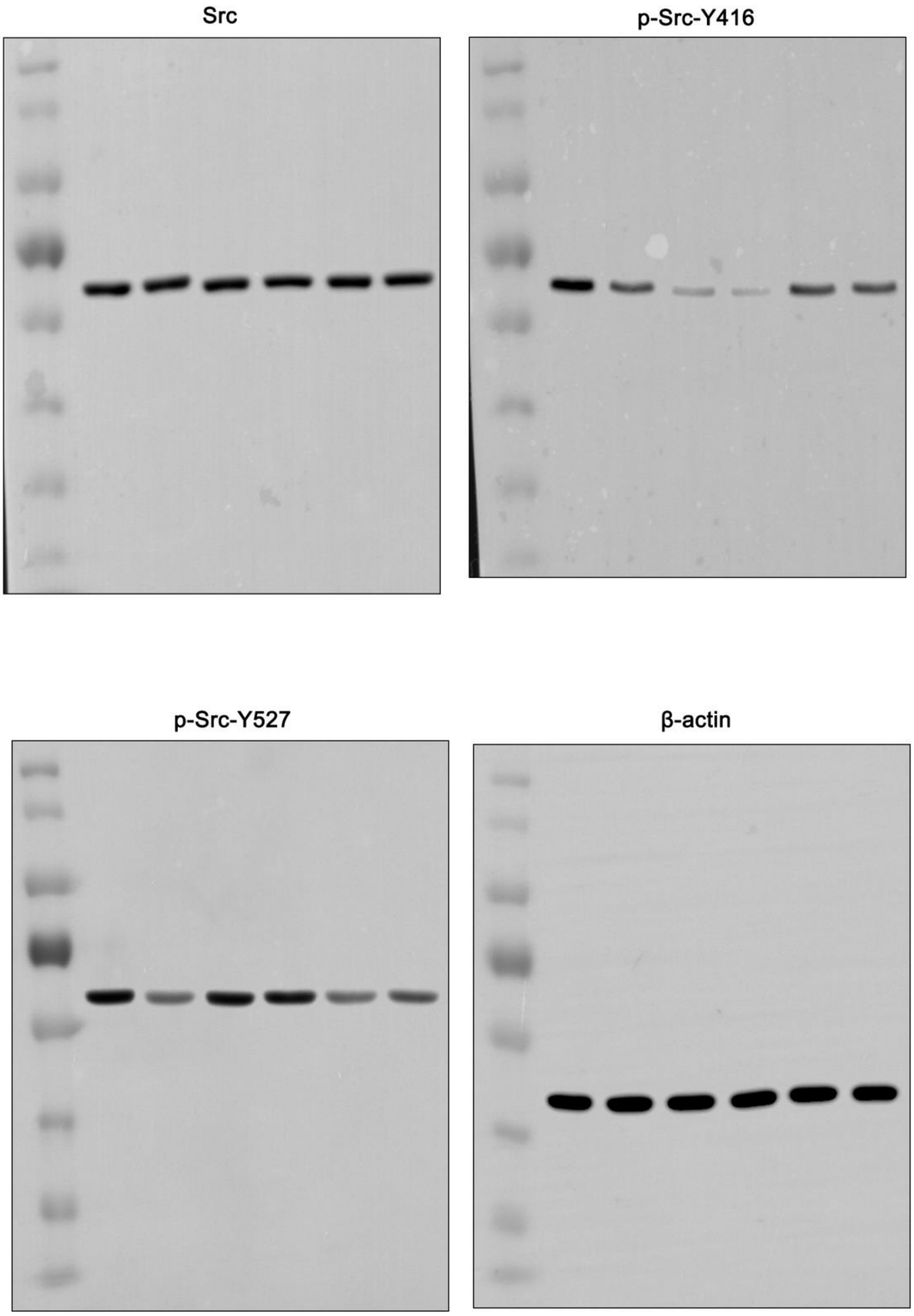

Supplementary Figure 4. Representative full-gel images of Western blots in Figure 5A.

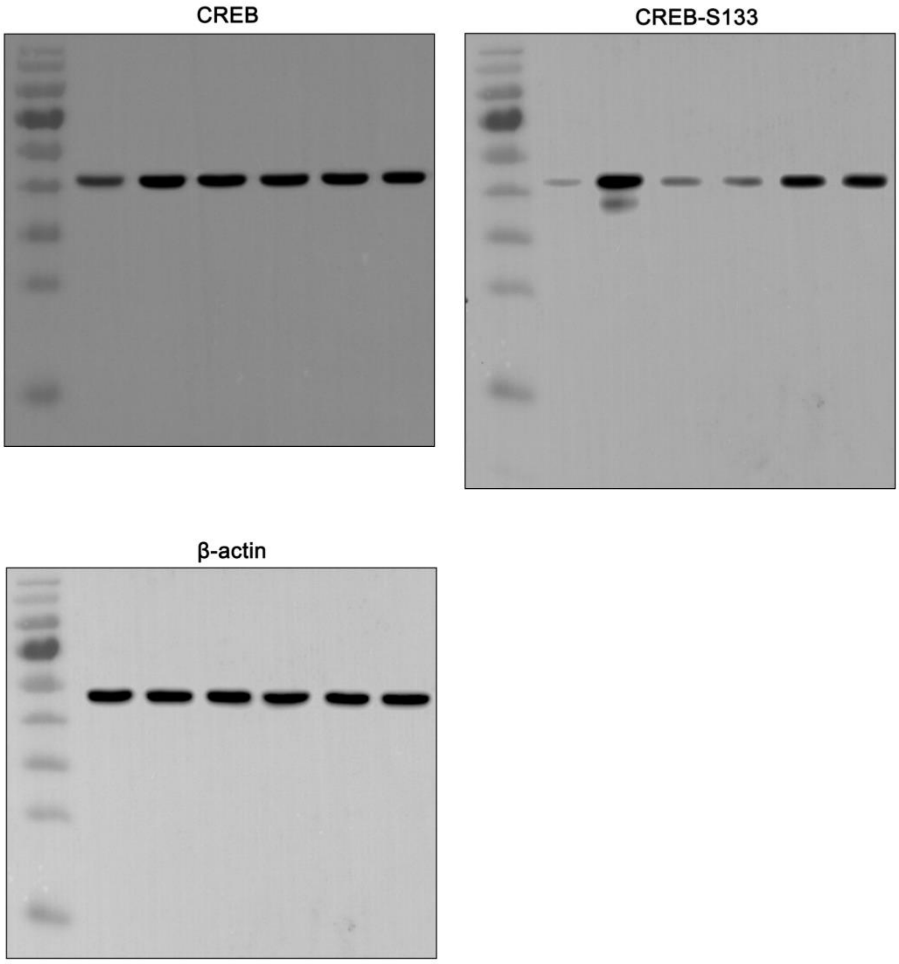

Supplementary Figure 5. Representative full-gel images of Western blots in Figure 6A.

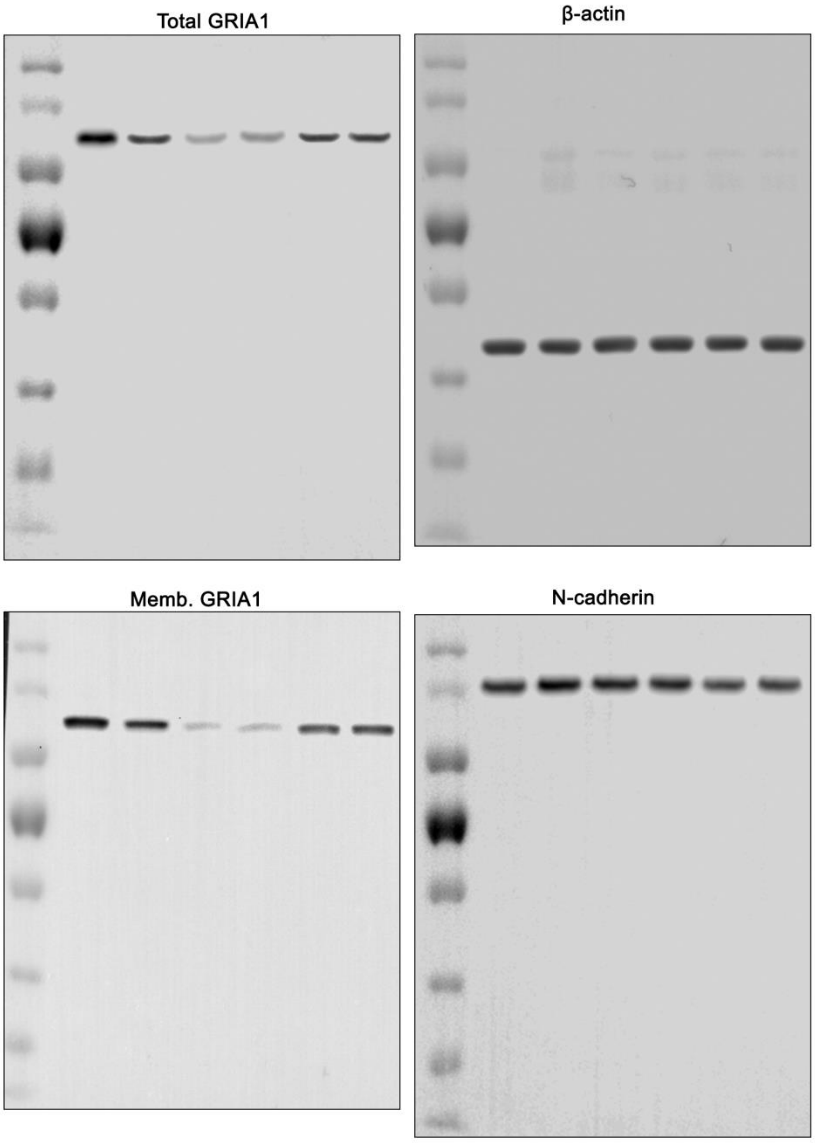

Supplementary Figure 6. Representative full-gel images of Western blots in Figure 7A.
